# Dairy Goat Farming in Alpine Mountain Areas: Sustainability and Profitable Approach

**DOI:** 10.3390/ani16121794

**Published:** 2026-06-10

**Authors:** Laura Franziska Flach, Emilio Sabia, Thomas Zanon

**Affiliations:** 1 Faculty of Agricultural, Environmental and Food Sciences, Free University of Bolzano, 39100 Bolzano, Italy; laurafranziska.flach@unibz.it; 2 Department of Agricultural, Forest, Food, and Environmental Sciences, University of Basilicata, 85100 Potenza, Italy

**Keywords:** mountain goat farming, life cycle assessment, small-scale farms, profitability

## Abstract

Dairy goat farming is a niche but relevant livestock system in alpine regions, yet its economic viability and environmental performance remain poorly quantified. This study assessed the relationship between profitability and environmental impacts in dairy goat farms in South Tyrol (Northern Italy). Data were collected from ten alpine dairy goat farms through on-farm interviews and accounting records and exploratorily analyzed using full-cost accounting and life cycle assessment (LCA). Given the small and purposive sample, all findings should be interpreted as preliminary and hypothesis-generating rather than statistically representative. Environmental impacts were evaluated from cradle to farm gate using two functional units: 1 kg of fat- and protein-corrected milk (FPCM) and 1 ha of agricultural land. Farm income per kg FPCM was highly variable, ranging from −€1.10 to €2.50, and depended strongly on herd size and subsidies. Average global warming potential was 2.96 ± 1.18 kg CO_2_ eq per kg FPCM, but farm rankings changed when impacts were expressed per hectare. Pearson correlation and linear regression analyses showed a significant positive relationship between income and greenhouse gas emissions (r = 0.80, *p* < 0.05), indicating a trade-off between economic and environmental performance. Enteric methane and energy use were the main contributors to climate impacts. Improving productivity per animal rather than expanding herd size appears to be the most promising strategy to enhance profitability while limiting environmental burdens.

## Simple Summary

Mountain livestock systems play an important role in maintaining rural economies, traditional landscapes, and agricultural biodiversity in alpine regions. Among these systems, dairy goat farming represents a small but increasingly relevant sector that operates under challenging environmental and economic conditions. However, limited information is available on the relationship between farm profitability and environmental performance in these systems. This study explored how economic results and environmental impacts interact in dairy goat farms located in South Tyrol (Northern Italy). Data were collected from ten alpine dairy goat farms through on-farm interviews and accounting records. The analysis combined full-cost accounting to assess farm profitability with life cycle assessment to quantify environmental impacts. Environmental performance was evaluated from cradle to farm gate using two functional units: 1 kg of fat- and protein-corrected milk and 1 ha of agricultural land. By integrating economic and environmental indicators, the study provides a framework to better understand sustainability trade-offs in mountain dairy goat systems and supports the development of strategies to improve the sustainability of dairy goat farming in mountainous regions.

## 1. Introduction

South Tyrol, Italy’s northernmost province, is characterized by mountainous terrain where livestock farming plays a central role in agricultural land use [1]. In recent years, dairy systems—particularly cattle-based production—have faced increasing economic, animal-welfare-related and environmental pressures, including declining milk output and growing scrutiny of housing systems [2,3]. Against this backdrop, dairy goat farming has gained attention as a potentially resilient option for mountain areas from both an environmental and an economic perspective, particularly for small-scale farms operating under structural constraints [4,5]. Goats are well adapted to steep topography and heterogeneous forage resources and are able to convert otherwise marginal vegetation into milk and meat, offering niche products and diversified income opportunities in alpine environments [6,7]. From an economic perspective, dairy goat farms are often characterized by small herd sizes, high labor inputs and a strong reliance on direct marketing and public support, making farm profitability particularly sensitive to management and structural decisions [5,8]. In the alpine region of South Tyrol, a substantial fraction of goat milk is processed into cheese and yogurt via local cooperatives, yet the delivered volume declined by 2.58% in 2024, and most goats are still raised predominantly for kid meat under extensive conditions, with only a minority kept for milk production [1,9]. Official statistics confirm that dairy goat herds represent only a small share of the regional livestock population compared with meat-oriented flocks, underlining the niche character of this sector [10]. Beyond provisioning functions, extensive goat systems provide ecosystem services of high relevance for alpine socio-ecological systems by maintaining open grasslands, limiting shrub encroachment and contributing to hazard mitigation and biodiversity conservation [11,12]. Preliminary evidence from this study indicates that farm income is on average positive only when public subsidies are included, while income excluding subsidies tends to be negative—a finding that underscores the importance of jointly examining economic viability and environmental performance in these systems. Despite this multifunctional role, dairy goat systems remain poorly represented in quantitative sustainability assessments, particularly with respect to the joint evaluation of economic viability and environmental performance in mountain regions. While the environmental performance of cow milk production in alpine contexts has been extensively assessed using life cycle assessment (LCA), including region-specific analyses for South Tyrol, comparable and integrated evidence for dairy goat systems is still limited, especially when economic outcomes and environmental impacts are analyzed simultaneously at farm level [13,14]. Methodological choices strongly affect result interpretation in grassland-based mountain systems, with the choice of functional unit (FU) being especially influential for ranking farms and production systems [15,16]. For dairy goat systems, the selection of an appropriate functional unit is particularly critical due to differences in milk composition and production characteristics compared with cattle, which can substantially influence LCA outcomes [17]. Concurrently, international guidance highlights the need for transparent and harmonized approaches to assess environmental impacts in small ruminant supply chains, particularly given the heterogeneity of extensive production systems [18]. Therefore, this preliminary study aimed to characterize alpine dairy goat farms in South Tyrol for the first time and to jointly assess their economic viability and environmental performance using full-cost accounting and life cycle assessment, explicitly comparing product-based (per kg fat- and protein-corrected milk, FPCM) and area-based (per ha) functional units to elucidate trade-offs relevant for farm management and policy design in mountain regions. This study should therefore be understood as a pilot investigation that provides a first quantitative characterization of dairy goat farms in South Tyrol. By capturing a range of structural farm types, it aims to deliver initial empirical evidence and a methodological framework, with the explicit purpose of informing future research based on larger and more representative samples.

## 2. Materials and Methodology

### 2.1. Study Design and Data Collection

The present study was conducted with the voluntary participation of ten dairy goat farms located in the alpine region of South Tyrol. To ensure confidentiality, all data were anonymized, and no identifying information was disclosed at any stage of the research. Farm selection was based on expert recommendations provided by the South Tyrolean Dairy Association [19], aiming to capture a representative sample of regional production systems. It should be noted that the total population of dairy goat farms specializing in milk production in South Tyrol is small, and the ten farms included in this study represent a purposive rather than random sample. Given this small absolute sample size, all quantitative analyses are therefore conducted in a strictly exploratory capacity. The sample encompasses a wide range of farm types with respect to herd size, land use, and management intensity (see Table 1), which, reflects the structural diversity of the sector. Consequently, all findings should be interpreted as exploratory and context-specific, and caution is warranted when drawing broader inferences. Farm visits in person took place in February 2025. During each visit, semi-structured interviews were conducted with the farm operators. These interviews addressed key aspects of farm management, including economic performance, structural characteristics, housing systems, and feeding practices. To evaluate the economic situation, farmers were asked to provide detailed records of all revenues and expenditures associated with milk production. Additionally, long-term investments were documented, specifically those related to buildings (over the past 25 years) and machinery (over the past 20 years). To assess labor input, farmers completed a structured questionnaire detailing their daily work routines and the time allocated to specific tasks within the barn. Occasional or irregular activities—such as hoof trimming or assistance during kidding—were also recorded. Labor time associated with forage production (e.g., haymaking) was excluded from the analysis. This exclusion was necessary because none of the participating farms maintained separate time records for forage-related activities, and retrospective attribution of working hours to haymaking and related tasks would have introduced unacceptable estimation uncertainty. The forage production cost model by [20] was therefore used as a standardized substitute, with the machinery component already accounted for in the fixed cost framework. Instead, forage production costs were estimated using the model developed by [20] which accounts for plot size, slope, and cutting frequency. Given that machinery costs had already been captured in the broader farm-level cost accounting framework (under fixed costs: machinery depreciation and upkeep), and given that [20] attribute approximately 50% of total forage production costs to machinery use, only the remaining 50% of the model-derived costs—representing non-machinery inputs such as labor and materials—were included in the forage cost calculation to avoid double-counting of machinery expenditures. This approach was chosen to avoid double-counting, since the machinery costs underlying forage production had already been captured within the farm-level depreciation and operational cost items. The exclusion of forage-related labor from the time-use analysis reflects the difficulty of separating this activity from other farm tasks in a consistent and verifiable manner across farms; it represents a simplification that may lead to a slight underestimation of total labor input, particularly on farms with large grassland areas [21]. Additionally, 5-point Likert scales were used to capture farmers’ perceptions regarding the relationship between work effort and farm income, the likelihood of continuing milk production over the next ten years, and their intention to adjust milk yield per goat in the near future. These responses provide further insights into the potential link between economic viability and the risk of farm discontinuation among dairy goat producers.

### 2.2. Calculation of Profitability

All economic data refer to the calendar year 2024 and are presented in euros (€) or euro cents (c). Since two farmers opted not to provide their economic data, the analysis of economic outcomes is limited to eight farms. A comprehensive full-cost analysis was performed for each individual farm, as well as aggregated across all farms. Both variable and fixed costs were considered in the analysis, alongside gross margin and net farm income. Variable costs, which are directly influenced by production volume, included expenditures for feed, veterinary services, stock replacement, bedding materials and transportation. Fixed costs, which are independent of production scale, encompassed depreciation of buildings and machinery, electricity and fuel, maintenance and repairs, insurance, and consultancy fees. The classification of costs followed the methodology outlined by [21]. Gross margin was calculated as the total revenue—comprising income from milk and animal sales minus variable costs. Net farm income was then derived by subtracting fixed costs from the gross margin. To facilitate cross-farm comparisons, all revenue and income figures were standardized per hectare of managed grassland, per number of dairy goats and per kg of fat- and protein-corrected milk (FPCM) produced. FPCM was calculated using the available milk yield data for the Saanen breed. As this was the predominant breed in all herds and no data were available for other breeds, FPCM was determined based on these records. The formula applied was taken from [17], and is particularly suitable for breeds with a milk fat content below 4%. It is stated as follows:

$$FPCM=M\cdot \left[\left(0.12\cdot FC+0.10\cdot PC+0.23\right)\right]$$

where

*FPCM* = fat- and protein-corrected milk (kg)

*M* = milk yield (kg)

*FC* = milk fat content (%)

*PC* = milk protein content (%)

For clarity and conciseness, only the most significant cost components are presented in detail while minor items are grouped under the category “others.”

### 2.3. Life Cycle Assessment

According to the [22,23] standards, the life cycle assessment (LCA) follows a structured framework consisting of four main phases: (1) definition of the goal and scope, including the identification of the functional unit and system boundaries; (2) life cycle inventory (LCI) analysis, which involves collecting data on all inputs and outputs of the processes considered; (3) life cycle impact assessment (LCIA); and (4) interpretation of the results obtained throughout the life cycle. In brief, the system encompasses all on-farm and upstream processes from feed production through to the farm gate, while infrastructure (buildings, machinery construction) and veterinary medicines are excluded. An economic allocation method was applied to distribute the farm’s greenhouse gas emissions between its primary output, milk, and the secondary co-product, kid goat meat. Allocation fractions were calculated based on farm-specific revenues: on average, 94.4% of total revenue was attributed to milk and 5.6% to kid goat meat sales (see Table 1 for farm-level values). Accordingly, 94.4% of total GHG emissions per farm were allocated to milk production and used as the basis for the per kg FPCM environmental impact results. Midpoint environmental impacts were evaluated using openLCA 2.5.0 in combination with the Agribalyse v3.0.1 database. The Environmental Product Declaration (EPD) method was applied for characterization. The characterization factors used were as follows: 1 CO_2_, 28 CH_4_ and 265 N_2_O kg CO_2_ eq respectively [24]. This approach provided standardized characterization factors for calculating global warming potential over a 100 y time horizon (GWP_100_) expressed in kg CO_2_ eq, acidification potential (AC) expressed in g SO_2_ eq, eutrophication potential (EP) expressed in g PO_4_ eq and water scarcity (WS) expressed in m^3^ eq across all farm-level inventories. The functional unit (FU) selected was 1 kg of FPCM produced by the lactating goats. Milk data from the Saanen breed, the predominant breed in the herds studied, were representative of the farms investigated for the calculation of FPCM. Additionally, given the huge variability in dairy goat farms, and in line with recent advances in the modeling of agricultural systems [25,26], a second FU was considered, namely one hectare (ha) of land. Using two functional units is essential in this context because product-based indicators (per kg FPCM) tend to favor high-yielding, input-intensive systems, whereas land-based indicators (per ha) better capture the environmental pressure of extensive systems with large land areas and low stocking densities [16]. The comparison of both units therefore allows trade-offs between production efficiency and land stewardship to be made explicit, which is particularly relevant for policy design in mountain regions.

This evaluation considered the total land of the studied farms to produce forages and concentrated feed. The system boundaries were defined as “cradle to farm gate” and encompassed all farm activities (on-farm and off-farm), except for machinery and building construction as well as veterinary medicines [18]. Total emissions were quantified for each farm system, accounting for enteric methane emissions, manure handling, energy use (fuel and electricity), management of permanent pastures, production of meadow hay, and the upstream production of concentrate feed. Methane emissions were calculated using coefficients provided by [27]. Methane (CH_4_) emissions associated with enteric fermentation, manure storage systems, and direct deposition on grasslands were estimated in accordance with the Tier 2 calculation approach formally recognized by the competent authority [28]. The methane conversion factor (Ym) was set at 5.5% for adult goats [28] in accordance with the IPCC (2019) Tier 2 default value for small ruminants under temperate conditions (IPCC 2019, Table 10.11 [28]). Feed quality digestibility was assumed at 65%, consistent with the extensive grassland-based diet typical of alpine goat systems and within the range specified by [28] for low-to-medium quality roughage diets. The feed quality digestibility was 65%. Methane emissions from manure were estimated based on a volatile solids (VS) excretion rate of 2.4 kg animal^−1^ day^−1^ and a maximum methane production potential of 0.1 m^3^ CH_4_ kg^−1^ VS. The methane conversion factor (MCF) was assumed to be 27% for pit storage systems and 1.5% for manure deposited on pasture. For calculation of direct N_2_O emissions from manure management the following equation was used [28]:

$$N_{2}O_{\left(mm\right)}=\left[\sum_{S}\left[\sum_{T,P}\left(N_{\left(T,P\right)}\times \;N_{ex(T,P)}\times {MS}_{\left(T,S,P\right)}\right)\right]\times {EF}_{3(S)}\right]\times \frac{44}{28}$$

where:
$N_{2}O_{\left(mm\right)}$= direct N_2_O emission from Manure Management in kg N_2_O yr^−1^;$N_{\left(T,\;P\right)}$= number of heads (T) in a productivity system (P);$N_{ex(P)}$= annual average N excretion per head (T) in a productivity system (P) in kg N animal^−1^ yr^−1^;${MS}_{\left(T,S,P\right)}$fraction of total annual nitrogen excretion for goats (T) that is managed in manure system S in the productivity class P, dimensionless;${EF}_{3(S)}$= emission factor for direct N_2_ = emissions from manure management system S in kg N_2_O-N/kg N in manure management system S;$\frac{44}{28}$= conversion of N_2_O-N_(mm)_ emissions to N_2_O_(mm)_ emissions.

The equation presented below was developed for the studied farms, which operated under a low-productivity system and with solid storage as their primary manure management strategy.

$$N_{2}O_{\left(mm\right)}=\left[\sum_{S}\left[\sum_{T,\;P}\left(N_{\left(T,P\right)}\times 16.2\;kg\;N\;{head\;}^{-1}{year}^{-1}\times 72\%\right)\right]\times 0.01\right]\times \frac{44}{28}$$

For indirect N_2_O emissions a coefficient of 2.33 kg head^−1^ year^−1^ was calculated, following the guidelines of [27]. Carbon dioxide (CO_2_) emissions attributable to energy use were quantified by considering both on-site emissions from fossil fuel combustion and off-site emissions related to electricity consumption. Estimates were derived from recorded diesel fuel use, expressed in liters, and electricity demand, expressed in kilowatt-hours, across the main farm activities. Consistent with the approach described by [27], a diesel density of 0.85 kg L^−1^ and a CO_2_-equivalent emission factor of 3.14 kg kg^−1^ of combusted diesel were applied. Emissions associated with electricity consumption were calculated using an Italy-specific factor of 0.47 kg CO_2_ eq kWh^−1^. An economic allocation was applied to distribute greenhouse gas emissions between milk and kid meat, using the revenue shares reported in Table 1.

### 2.4. Statistical Analysis

Given the limited and exploratory nature of this pilot study (n = 8 for economic analyses, n = 10 for LCA), no confirmatory statistical inference is attempted. All Pearson correlation coefficients, regression slopes, and associated *p*-values are reported descriptively to identify potential patterns and generate hypotheses for future studies with adequate statistical power. Stepwise variable selection was applied to reduce overfitting, but the resulting model should be regarded as exploratory. Collinearity diagnostics (tolerance and variance inflation factor) were performed to assess the stability of the final models. This approach was chosen to reduce the risk of overfitting and to ensure model parsimony. Furthermore, to examine the relationship between economic performance and environmental impact in dairy production in terms of GWP_100_, a linear regression analysis was conducted. The dependent variable was GWP_100_ per kilogram of FPCM while the independent variable was income per kilogram FPCM including subsidies. Additionally, Pearson correlation coefficients were calculated to explore associations between income and multiple environmental indicators, including AC, EC and WS. All analyses were done using IBM Statistics SPSS 29.

## 3. Results

### 3.1. Farm Characteristics

The surveyed dairy goat farms were located in mountainous regions between an altitude of 900 and 1920 m a.s.l. and showed considerable variation in herd size (Ø 82 ± 97) lactating goats per farm), milk yield, and feeding intensity. In total, 90% of farms operated under loose housing systems and provided pasture access during the vegetation period. In total, 60% of farms were managed full-time and engaged in direct marketing. However, 75% of the part-time farms sold their products directly as well. Farms were mainly managed conventional while organic certification was only present in two farms. Table 2 shows the characteristics of the studied farms. Overall, the sample reflects a diverse range of production conditions typical for alpine dairy goat farming.

### 3.2. Farm Profitability

Farm income including subsidies is positive on average, while income without subsidies tends to be negative. Revenues are primarily generated through milk sales, with additional contributions from subsidies and sold animals. Variable costs per farm and year are moderate, with forage production and concentrate feed representing the largest shares. On average, fixed costs amount to €46,765 and are therefore nearly four times higher than variable costs (see Table 1). Workload per day and year is relatively consistent across farms. Positive values for payment per working hour can only be observed when subsidies are included. Although economic performance was standardized both per kilogram of FPCM (Table 3) and per hectare (Table 4), variability was observed across farms. In both units of analysis, fixed costs clearly exceeded variable costs, resulting in negative net income for several farms—particularly when subsidies were excluded from the calculation. However, high standard deviations show that there is substantial variation in income and cost structures across farms.

### 3.3. Farmers Attitudes

Table 5 indicates that most farmers are dissatisfied with the balance between workload and remuneration, with 50% responding “rather not” or “absolutely not” when asked about their satisfaction. Despite this, 60% of respondents expressed absolute certainty that they will continue milk production over the next ten years. Regarding future milk yield per goat, 20% intend to decrease production; another 20% plan to maintain current levels, while the remaining 60% aim to significantly increase milk output. This is also reflected in farmers’ breeding strategies, which are shown in Table 6.

### 3.4. Factors Influencing Farm Income

Variables such as grassland area, working hours per day and concentrate feed per goat showed a significant correlation with farm income per kg FPCM but were excluded from the final model due to lack of statistical contribution. Thus, the remaining independent variable in the model was the number of dairy goats. The model explained 47.1% of the variance in income (adjusted R^2^ = 0.383). Given the small sample size (n = 8), the *p*-value is reported for descriptive completeness only and should not be interpreted as confirmatory evidence. The unstandardized coefficient indicated that each additional dairy goat was associated with an increase of approximately €162.88 in farm income.

### 3.5. Environmental Performance

**Figure 1:**
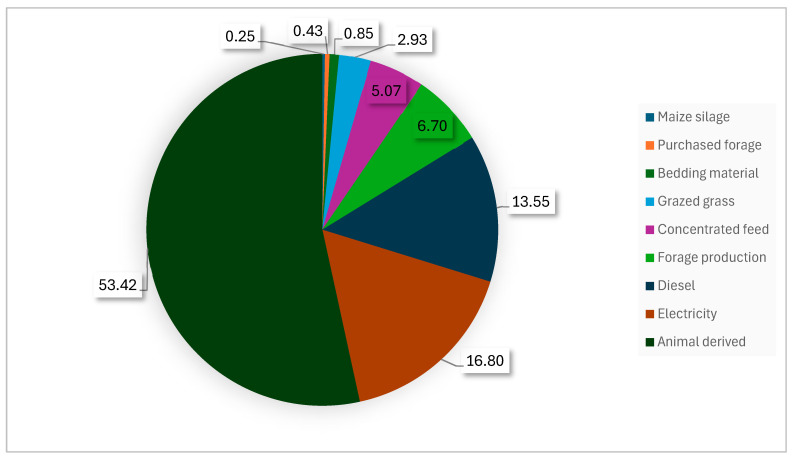
Main process contributors on GWP_100_ (kg CO_2_ eq) in %.

Table 7 shows the environmental results for 1 kg of FPCM goat milk and per ha of farmland. The average GWP_100_ of the South Tyrolean dairy goat farms analyzed in this study was 2.96 ± 1.18 kg CO_2_ eq per kg FPCM (mean ± SD). For instance, one farm that shows the lowest emissions per kg FPCM, with 1.66 kg CO_2_ eq, ranks only slightly below average when emissions are expressed per hectare, with 5315 kg CO_2_ eq. Conversely, the farm with the highest emission value per kg of FPCM (4.79 kg CO_2_ eq) exhibits the second-lowest emission value per hectare, at 3137 kg CO_2_ eq. The impact of the chosen FU is also evident in the AC results. While one farm showed the lowest value per kg FPCM, it ranked second highest when emissions were expressed per hectare. In contrast, EC results were only slightly affected by the change in FU; farms generally remained within their respective low, medium, or high categories. For WS, the farm with the highest and the one with the lowest values per kg FPCM retained their respective positions when the FU was changed to per hectare. Other farms showed minor shifts in ranking. Independent of the chosen functional unit, the standard deviations indicate a high variability in environmental impacts. Figure 1 presents the main contributors to GWP_100_, showing that emissions directly attributable to the animals account for more than half of total GWP_100_. Diesel and electricity together contribute to more than one third, while the remaining share is associated with feed production. The AP was primarily driven by four processes: diesel use (31%), electricity consumption (30%), forage production (15%) and grazing (14%). EP was mainly caused by four sources: forage production (31%), grazing (26%), electricity consumption (12%), and concentrate feed (11%). WS was largely attributed to concentrate feed, contributing 45%, followed by electricity consumption with 40%.

### 3.6. Relationship Between Economic and Environmental Performance

**Figure 2:**
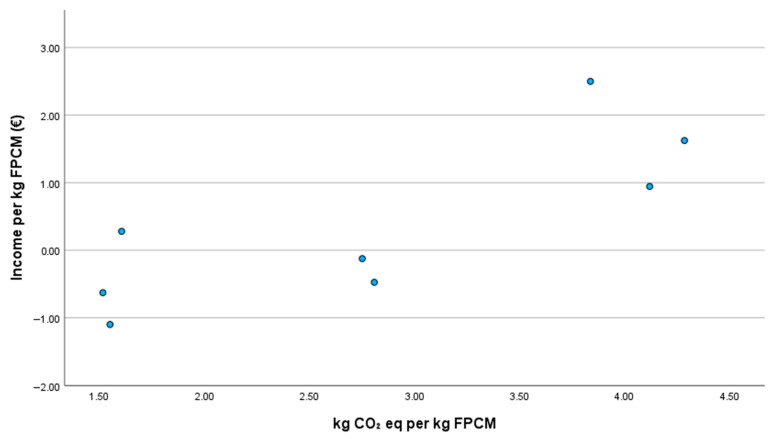
Income per kg FPCM (incl. subsidies) in relation to CO_2_-equivalent emissions.

A strong positive correlation was observed between income per kg FPCM and GWP100 per kg FPCM (r = 0.799). Given the exploratory nature of this analysis (n = 8), this association should be interpreted as a preliminary pattern rather than a statistically robust finding. Positive trends were also observed for AC per kg FPCM (r = 0.672, *p* = 0.068), EC per kg FPCM (r = 0.608, *p* = 0.110), though these did not reach statistical significance. A simple linear regression (exploratory) suggested that for each additional euro of income per kg FPCM, CO_2_ eq emissions increased by approximately 0.77 kg per kg FPCM (R^2^ = 0.639, n = 8). This relationship requires confirmation in larger samples. Figure 2 shows the relationship between income per kg FPCM and GWP_100_ per kg FPCM on farm level.

## 4. Discussion

### 4.1. Farm Structure

Even though approximately 70% of dairy farms in South Tyrol operate on a part-time basis [19], only 40% of the farms included in our study fell into this category. This discrepancy is likely related to the high proportion of direct marketers among the surveyed farms, which contrasts with the overall share of 2.8% of direct marketing farms in South Tyrol [29]. This suggests a potential selection bias resulting from the voluntary nature of participation. Given the small and purposive sample of ten farms, structural observations reported here should be considered descriptive and illustrative of the sector’s diversity rather than statistically representative of alpine dairy goat farming in South Tyrol. While tie-stall housing remains common in dairy cattle systems in South Tyrol [30], this housing type is generally not used in dairy goat farming, as current regulations and species-specific welfare requirements prohibit the tethering of goats. Compared to cattle, the structural requirements for loose housing for goats are substantially lower, reducing construction costs and facilitating implementation for most farmers [31]. Loose housing combined with pasture access also tends to align with consumer expectations, as many consumers prioritize animal welfare over environmental performance when choosing dairy products [32]. Herd sizes in the analyzed farms were generally small, although considerable variation was observed. In contrast, more intensive goat farms in Lombardy tend to have substantially larger herds and significantly higher milk yields, averaging 711 kg FPCM per goat and year, which is approximately twice the yield observed in this study. These systems, however, rely on more than twice the amount of concentrate feed [33,34]. Higher milk yields have also been reported for low-input grazing systems in New Zealand (458 kg FPCM), achieved with very low levels of concentrate supplementation, indicating that productivity improvements are possible without full intensification. Conversely, even lower yields are reported for low-input systems in Greece, averaging 175 kg per goat and year [35], underlining the strong influence of regional conditions. On average, the farms managed approximately 12 ha of grassland per holding and operated at livestock densities below 1 LU ha^−1^, classifying them as extensive systems. From an ecological perspective, extensive farming systems are known to provide a broad range of ecosystem services, including the maintenance of open grasslands and landscape heterogeneity [36,37]. Recent syntheses highlight that small ruminants provide a wide range of critical ecosystem services in mountain regions, including the maintenance of open grasslands, prevention of shrub encroachment, and support of biodiversity-rich habitats [38]. Beyond their productive function, these systems play an essential role in sustaining cultural landscapes, preserving ecological functions, and delivering socio-cultural values in alpine environments characterized by strong natural constraints.

### 4.2. Economic Performance

The full-cost analysis revealed considerable heterogeneity among the analyzed farms. The pattern of subsidy-dependent viability—with several farms remaining unprofitable even with public support—points to structural rather than managerial shortcomings, consistent with findings from comparable mountain livestock systems [39].

Similar patterns have been reported for dairy goat farms in Bulgaria, where only one out of eight farms achieved a positive income without subsidies [8]. However, this phenomenon is not unique to goat farming. Comparable economic challenges have been documented for small-scale dairy cattle farms in South Tyrol, where income per hour of labor remains negative or very low even when subsidies are considered [2]. This aligns with broader evidence from alpine livestock systems: small farms in mountainous areas frequently depend on direct payments to offset structurally higher production costs, with competitiveness hinging on sustained policy support [39]. Beyond simple scale effects, the observed economic performance is largely driven by the interaction between structural constraints and recent investment decisions. Many of the analyzed farms have invested in new housing facilities and machinery to comply with animal-welfare standards and to ensure long-term sustainability. While these investments are rational from both a strategic and ethical perspective, they generate high depreciation costs that are difficult to offset given the low production volumes typical of mountain goat systems. As a result, cost structures are dominated by fixed costs, making profitability particularly sensitive to herd size, labor efficiency and capital intensity. It should be noted, however, that the economic patterns described here are based on eight farms and should be interpreted with caution; the high variability observed across farms further limits the extent to which these findings can be generalized. In alpine case studies, high fixed costs—especially building and machinery depreciation—consistently dominate cost structures and exert downward pressure on operating results, reinforcing the need for cautious investment planning (e.g., shared facilities and machinery) [40]. The high variability in farm income—ranging from −€1.10 to €2.50 per kg FPCM—is not simply a reflection of differences in scale but also of heterogeneous investment histories, marketing strategies, and subsidy uptake. Farms that rely heavily on subsidies to achieve positive income face a structural vulnerability: any reform of area-based or coupled support payments under the Common Agricultural Policy (CAP) could rapidly render these operations economically unviable. This dependency raises broader questions about the long-term sustainability of public support as the primary income stabilizer for mountain goat systems, and points to the need for complementary strategies—such as differentiated market positioning, direct marketing, and improved productivity per animal—that reduce exposure to policy risk [39,41]. Milk production in mountainous regions is generally associated with higher production costs than in lowland areas [42], a pattern clearly reflected in this study. Production costs ranged from €0.98 to €3.81 per kg FPCM, exceeding reported values for goat milk production in Germany (€1.11–€1.34 depending on herd size) [43]. Herd size emerged as the only variable with a significant influence on farm income. However, increasing herd size is particularly challenging in mountain regions, where space is limited, forage availability is constrained, and stocking density thresholds are tied to subsidy eligibility [41]. These constraints restrict the ability of farms to exploit economies of scale, placing small-scale mountain farms at a structural disadvantage, as also observed in other agricultural sectors [2,44]. Despite the economically challenging situation, the substantial investments made by the surveyed farmers indicate a strong intention to continue milk production. Most respondents stated that they expect to still be producing milk in ten years, even though they expressed dissatisfaction with their current remuneration. Furthermore, the fact that 50% of respondents plan to increase milk yield suggests an attempt to compensate for unfavorable economic conditions through intensification and higher productivity rather than structural expansion.

### 4.3. Environmental Performance and Climate Impacts

The environmental performance of the analyzed dairy goat farms is strongly shaped by their extensive management, grass-based feeding systems and low stocking densities. Among the assessed impact categories, climate change, expressed as GWP_100_, represents a particularly relevant dimension given the prominence of greenhouse gas emissions in sustainability debates surrounding ruminant livestock production. The average GWP_100_ amounted to 2.96 kg CO_2_ eq per kg FPCM. Comparable values have been reported for intensive dairy goat farms in Lombardy (2.67 kg CO_2_ eq kg^−1^ FPCM), whereas pasture-based systems in New Zealand showed substantially lower impacts (1.03 kg CO_2_ eq kg^−1^ FPCM), reflecting differences in productivity, feeding strategies and system boundaries [33,34]. Studies accounting for carbon sequestration effects reported even lower climate impacts for grazing systems [45], illustrating the sensitivity of climate results to methodological choices. When compared with dairy cow systems, goat milk production generally exhibits higher GWP_100_ values [46]. Differences in milk composition and yield characteristics between goats and dairy cows further contribute to these discrepancies and underline the importance of appropriate standardization when comparing ruminant milk products on a per kg basis [17]. However, the minimum value observed in this study (1.52 kg CO_2_ eq kg^−1^ FPCM) demonstrates that individual goat farms can achieve climate performance levels comparable to those reported for extensive dairy cow farms in South Tyrol [47]. The relatively higher climate impact per kilogram of FPCM observed in many farms is primarily a consequence of low productivity per animal rather than inefficient management. In extensive mountain systems, limited forage quality, short vegetation periods and restricted concentrate use constrain milk yield potential. As enteric fermentation emissions are largely linked to animal maintenance requirements, lower milk output per goat leads to higher emission intensities per unit of product, even when absolute emissions per animal remain moderate. This is consistent with the broader LCA literature for small ruminants: Gutiérrez-Peña et al. [45] demonstrated that improving milk yield per animal through optimized grazing management substantially reduced carbon footprint per kg FPCM without increasing stocking density, and Zucali et al. [34] showed similar effects in intensive Italian systems through dietary and genetic improvements.

These findings are, however, derived from a limited sample of ten farms, and the observed range of environmental impacts reflects the structural heterogeneity of the sample rather than a statistically robust characterization of the sector. Beyond climate change, the environmental profile was further shaped by acidification, eutrophication and water scarcity impacts. Acidification potential was comparable to values reported for dairy goat farms in France [48] and fell within the range reported for European dairy cow systems [13,49]. Major contributors included diesel use, electricity consumption, forage production and grazing, reflecting the mechanization effort required under steep alpine conditions. Eutrophication impacts were mainly driven by nutrient losses associated with manure deposition during grazing, while water scarcity impacts were strongly influenced by the reliance on imported concentrate feed produced in water-scarce regions.

### 4.4. Influence of Functional Unit Choice on Environmental Assessment

The choice of functional unit plays a decisive role in the interpretation of environmental impacts, particularly in heterogeneous livestock systems characterized by strong contrasts between productivity and land-use intensity. When environmental impacts were expressed per hectare, the analyzed farms showed substantially lower impacts than intensive goat farms in Lombardy, largely due to their low stocking rates and extensive land base [34]. These findings underscore the critical role of functional unit selection in environmental assessments of livestock systems and are consistent with earlier LCA studies highlighting that different functional units can lead to contrasting sustainability rankings [50]. Ref. [16] demonstrated that product-based indicators tend to favor high-yielding systems, whereas area-based indicators better capture land use efficiency and environmental pressure in extensive systems. In the specific context of mountain agriculture, a land-based functional unit captures system performance more adequately than a product-based indicator alone. Land availability represents the primary limiting factor for farm development, while the maintenance of open grassland is a central policy objective. Assessing impacts per hectare therefore reflects both ecological pressures and land stewardship functions, whereas product-based indicators predominantly reward output intensity, which is structurally constrained in mountain regions. International guidelines similarly emphasize the need for transparency and consistency in functional unit selection, particularly for heterogeneous small ruminant systems, to ensure comparability and policy relevance [18]. While the functional unit comparison conducted here illustrates these dynamics clearly, the conclusions remain exploratory given the small sample size and should be validated in future studies with larger and more diverse farm populations.

### 4.5. Trade-Offs Between Economic Performance, Production Efficiency and Ecosystem Services

The results of this study highlight a fundamental tension between economic performance and environmental sustainability in alpine dairy goat systems. The significant positive relationship between farm income and GWP_100_ (r = 0.80, *p* < 0.05) suggests that farms achieving higher income tend to generate greater environmental burdens per unit of land. This trade-off arises primarily because income gains in the analyzed systems are achieved through herd expansion rather than productivity improvements per animal. Larger herds increase total milk output and revenue but simultaneously raise absolute greenhouse gas emissions, particularly from enteric fermentation [51]. In contrast, options to increase milk yield without adding animals remain underdeveloped, largely due to limited breeding infrastructure and suboptimal forage quality [52]. As a result, economic improvement and emission reduction currently follow opposing trajectories in many mountain goat farms. All regression and correlation analyses in this study are based on a sample of n = 8 (economic) and n = 10 (LCA) farms. This sample size is insufficient for confirmatory statistical inference; regression coefficients and Pearson r values are thus reported as descriptive indices of association and as a basis for hypothesis generation in future studies. No causal interpretation is warranted from these data alone.

This trade-off becomes particularly relevant when considered alongside the non-productive ecosystem services delivered by extensive alpine goat systems. Extensive goat farming in mountain regions contributes to the maintenance of open grasslands, limitation of shrub encroachment, hazard mitigation, and biodiversity conservation [53,54,55]. These services, however, are not captured in product-based economic indicators and are therefore systematically undervalued in conventional cost–benefit analyses [56]. Farms with lower milk output per animal—which, from a purely product-based LCA perspective, appear environmentally less efficient—may simultaneously deliver higher per-hectare ecosystem service values [57]. This is consistent with findings from other extensive small ruminant systems in European mountain regions, where agri-environment scheme participation correlates positively with landscape heterogeneity but negatively with milk productivity [53].

In the context of the Common Agricultural Policy (CAP), area-based and coupled support payments partially compensate farms for the provision of these landscape and environmental services [58]. In our sample, subsidies were a critical determinant of farm viability, particularly for part-time and low-intensity producers. The per-hectare LCA functional unit employed in this study partially captures this dimension: when environmental impacts are expressed per ha rather than per kg FPCM, farm rankings shift considerably, illustrating that the choice of functional unit is not merely a methodological decision but reflects an underlying value judgment about what constitutes ‘good’ farming performance [59,60]. For alpine dairy goat systems, which operate at the intersection of food production, landscape stewardship, and rural livelihoods, neither a purely product-based nor a purely area-based perspective is sufficient in isolation [61]. From a practical management perspective, the most tractable pathway to improving the income–emissions trade-off is increasing milk yield per animal through improved forage quality, targeted supplementation, and systematic selection for milk traits—an approach that reduces emission intensity without requiring herd expansion or additional land [34,43]. Policy instruments should reward this pathway explicitly, for instance through productivity-linked supplements within agri-environment schemes, rather than relying solely on area-based payments that inadvertently reward low-output systems.

Future assessments should integrate quantitative ecosystem service indicators—such as plant species richness, shrub cover dynamics, or erosion risk indices—alongside economic and LCA metrics, enabling a more comprehensive evaluation of farm sustainability that reflects the multifunctional character of alpine goat systems. Policy instruments should be designed accordingly, rewarding farms not only for production efficiency but also for the public goods they generate, particularly where market mechanisms fail to compensate for these services.

### 4.6. Limitations and Outlook for Future Research

This study is subject to some limitations that must be considered when interpreting the results. Most importantly, the sample size of ten farms—eight of which provided complete economic data—substantially limits statistical power. The significant correlation between farm income and GWP_100_ must therefore be treated as exploratory, as results from small samples are inherently sensitive to individual farm characteristics and outliers. This restriction reflects the limited population of specialized dairy goat farms in South Tyrol and the voluntary nature of participation but nonetheless precludes generalization beyond the study context. Additionally, all economic and management data relied on farmer self-reporting during on-farm interviews. While consistency checks were applied where possible, this approach is subject to recall bias and social desirability effects, which may particularly affect labor time estimates and cost reporting. Future studies should complement self-reported data with objective farm records or standardized accounting systems to improve data reliability. Furthermore, the sample exhibits considerable structural heterogeneity in herd size, land area, labor organization, and marketing strategy, including a relatively high share of part-time producers. While this mirrors the actual diversity of the sector, it complicates cross-farm comparisons and regression-based inference. The findings should consequently be interpreted as context-specific tendencies rather than representative patterns or causal relationships. Additionally, several dimensions relevant to a comprehensive sustainability assessment—including animal welfare and social indicators—were beyond the scope of this study and will be addressed in subsequent work. The sustainability evaluation presented here is therefore partial by design.

This study is explicitly conceived as a pilot investigation, with primary contributions that are methodological and descriptive. Future research should build on this foundation using larger, stratified samples, standardized accounting protocols, and, where feasible, longitudinal data collection to generate a more robust evidence base for sustainability-oriented policy in alpine mountain regions.

## 5. Conclusions

Our findings reveal a high degree of heterogeneity among dairy goat farms in the alpine region of South Tyrol. Most farms included in the study were engaged in direct marketing, and most goats were kept in loose housing systems with access to pasture. This type of management aligns more closely with current consumer and retail expectations than the tie-stall systems still commonly used in alpine dairy cattle farming. From an economic perspective, the results reflect a pattern observed in other small-scale farms: disproportionately high fixed costs significantly reduce profitability, and the limited farm size prevents producers from benefiting from economies of scale. On average, goat farms are not able to compete economically with dairy cattle farms in the region. However, individual cases demonstrate that under favorable conditions, goat farming can be a viable alternative. Despite the challenging economic situation and a strong reliance on subsidies, most farmers expressed a clear intention to continue milk production. When environmental impacts are assessed using different functional units, results vary considerably. In alpine regions like South Tyrol, where land availability is a limiting factor, expressing impacts per hectare offers a more accurate representation of overall resource use. Compared to dairy cattle farms, goat farms show a substantially lower environmental footprint when evaluated on a per-hectare basis. Herd size emerged as a key factor influencing both farm income and GWP_100_. Farms with higher income levels also tended to exhibit higher greenhouse gas emissions, primarily due to increased enteric fermentation. This trade-off should be carefully considered in the design of subsidy schemes to avoid unintended incentives that favor emissions-intensive practices. It must be emphasized that the patterns identified in this pilot study are based on a small, purposive sample and cannot be considered statistically generalizable to the wider population of alpine dairy goat farms. They should be understood as directional hypotheses requiring validation in larger, stratified samples. Within this limitation, the following implications appear plausible. Improving farm profitability without increasing herd size will require targeted strategies to enhance individual animal performance. This includes the development of structured breeding programs, optimized feeding strategies, and improved management practices. Currently, such approaches are largely underdeveloped in South Tyrol’s goat sector, highlighting an urgent need for further research and innovation.

## Figures and Tables

**Table 1:** Description of farms. (N = 10).

Item	Mean	Minimum	Maximum
Meter a.s.l	1408 (281)	900	1920
Lactating goats, no.	82 (97)	28	350
Kid goats, no.	32 (27)	0	80
Livestock units/ha	0.93 (0.29)	0.53	1.52
FPCM/goat and year, kg	356.70 (151)	172	541.60
Full time, %	60		
Direct marketing, %	70		
Organic, %	20		
Permanent pasture access during vegetation period, %	80		
Grassland, ha	12 (11)	5	33.80
Concentrate/goat and day, kg	0.50 (0.4)	0	1.30

Meter a.s.l.: meter above sea level; FPCM: fat- and protein-corrected milk. Means; standard deviation in brackets.

**Table 2:** Revenues, costs and income in € (N = 8).

Item	Mean	SD	Min	Max
**Revenues**	64,256.56	71,999.92	16,176.64	239,034
Milk	44,520.73	56,834.91	11,300	183,391
Sold goats (milk goats, young goats and kid goats)	2654.43	1529.61	271	5370
Subsidies	13,965.08	9273.76	2811.64	27,787
Others	3940.71	10,426.15	0	27,585
Variable costs	11,967.19	9633.09	715	33,115
Feed	1936.43	2113.07	0	4695
Forage production	8822.357	5756.07	5500	18,650
Veterinary	1300	2168.11	200	6450
Stock replacement	700	1739.25	0	5000
Other	1424.29	2918.97	0	8015
Gross Margin	72.14	24.54	31.17	98.07
Fixed costs	46,765.42	49,386.69	2250	15,3101.7
Machinery depreciation	11,860.42	16,339.93	2000	51,166.67
Building depreciation	19,070	17,692.75	1560	40,000
Machinery and building upkeep	1262.5	1914.56	0	5000
Insurance	1756.13	2193.48	900	6504
Fees and consulting	7655.75	17,022.22	90	49,581
Power fuel	3134.29	2544.35	1750	7200
Wage labor	1381.25	3906.77	0	11,050
Other	1445.88	2963.29	0	8650
Summed costs	58,732.6	56,623.12	16,750	186,216.7
Income				
excl. Subsidies	−8441.13	21,649.3	−45,307	25,030.33
incl. Subsidies	5523.95	25,781.68	−35,731	52,817.33
Workload (h)				
per year	2386.29	650.75	1456	3362.9
per day	6.47	1.8	4	9.1
Payment/working hour				
excl. subsidies	−4.34	10.19	−20.74	11.06
incl. subsidies	2.75	12.01	−16.36	25.37

**Table 3:** Revenues, costs and income per kg FPCM in € (N = 8).

	per kg FPCM			
	Mean	SD	Minimum	Maximum
**Revenues**	2.81	1.53	1.26	5.44
Milk	1.73	0.73	0.97	2.83
Sold goats	0.26	0.22	0	0.68
Subsidies	0.83	0.7	0.15	2.28
Variable costs	0.72	0.66	0.09	1.87
Fixed costs	1.71	0.89	0.29	2.82
Summed costs	2.43	0.86	0.98	3.81
Income				
Income excl. subsidies	−0.45	0.85	−1.39	1.2
Income incl. subsidies	0.38	1.23	−1.1	2.5

**Table 4:** Revenues, costs and income per ha in € (N = 8).

	per ha			
	Mean	SD	Min	Max
**Revenues**	4290.49	1753.98	1841.08	7610
Milk	2895.59	1459.02	979.66	5520
Sold goats	323.38	244.65	6.05	716
Subsidies	1034.97	426.09	562.33	1613.79
Variable costs	1136.09	953.39	95.33	2614.5
Fixed costs	3166.25	2460.56	281.25	7027.91
Summed costs	4302.33	2800.35	1963.84	9399.7
Income				
Income excl. subsidies	−1046.83	1868.19	−4118.82	1261.93
Income incl. subsidies	−11.8514	1886.06	−3248.27	2633.13

**Table 5:** Farmers’ views on economic well-being and future farm decisions (N = 10).

	Mean (SD)	Min	Max
Do you agree/think that…			
relation of payment to workload is good? ^1^	3.2 (1.2)	1	5
you will still produce milk in 10 years? ^1^	1.6 (0.8)	1	3
you will change milk yield per goat? ^2^	3.7 (1.1)	2	5

^1^ 5-point-Likert scale from 1 = “Yes, absolutely” with 3 = “Partly” to 5 = “No, absolutely not”. ^2^ 5-point-Likert scale from 1 = “Much less milk” with 3 = “Equal milk yield” to 5 = “Much more milk”.

**Table 6:** Farmers’ breeding strategy (N = 10).

Which Trait Do You Consider Most When Selecting the Breeding Buck?	
Milk yield	50%
Milk components	10%
Body condition	40%

**Table 7:** Environmental impact indicators per kg FPCM and per ha (N = 10).

		per kg FPCM				per ha			
Impact categories	Unit	Mean	SD	Min	Max	Mean	SD	Min	Max
Global warming potential (100)	kg CO_2_ eq	2.96	1.18	1.66	4.79	5427	1882	2994	9698
Acidification potential	g SO_2_ eq	12.6	5.2	10.1	20.4	23,740	10,480	13,150	49,210
Eutrophication potential	g PO_4_ eq	9.9	7.2	0	31.7	17,020	7240	9720	27,780
Water scarcity	m^3^ water eq	1.1	0.6	0.5	2.1	2573	2738	511	9975

## Data Availability

Data will be made available on request.
